# Accumulation of minor alleles and risk prediction in schizophrenia

**DOI:** 10.1038/s41598-017-12104-0

**Published:** 2017-09-15

**Authors:** Pei He, Xiaoyun Lei, Dejian Yuan, Zuobin Zhu, Shi Huang

**Affiliations:** 1 Laboratory of Medical Genetics, School of life sciences, Central South University, 110 Xiangya Road, Changsha, Hunan 410078 China; 2 Department of Genetics, Xuzhou Medical University, Xuzhou, Jiangsu 221004 China

## Abstract

Schizophrenia is a common neuropsychiatric disorder with a lifetime risk of 1%. Accumulation of common polygenic variations has been found to be an important risk factor. Recent studies showed a role for the enrichment of minor alleles (MAs) of SNPs in complex diseases such as Parkinson’s disease. Here we similarly studied the role of genome wide MAs in schizophrenia using public datasets. Relative to matched controls, schizophrenia cases showed higher average values in minor allele content (MAC) or the average amount of MAs per subject. By risk prediction analysis based on weighted genetic risk score (wGRS) of MAs, we identified an optimal MA set consisting of 23 238 variants that could be used to predict 3.14% of schizophrenia cases, which is comparable to using 22q11 deletion to detect schizophrenia cases. Pathway enrichment analysis of these SNPs identified 30 pathways with false discovery rate (FDR) <0.02 and of significant P-value, most of which are known to be linked with schizophrenia and other neurological disorders. These results suggest that MAs accumulation may be a risk factor to schizophrenia and provide a method to genetically screen for this disease.

## Introduction

Schizophrenia is one of the most frequent neuropsychiatric disorders with a lifetime risk of 1% in the general population^1, 2^. This disease is often chronic and places a great burden on family and society. It is characterized by the occurrence of delusions, hallucinations, disorganized speech and behavior, impaired cognition, and mood symptoms^3^. Data from twin, family, and adoption studies provide strong evidence that schizophrenia is a genetic disorder with high heritability^4^.

The precise mode of schizophrenia inheritance is unclear and risk prediction using known genetic components is presently unrealistic. Based on investigating familial syndromes with schizophrenia-like phenotypes, two rare variants have been identified as associated with schizophrenia: the 22q11 deletion^5, 6^ and a 1:11 translocation^7^. With the advent of copy number variants (CNVs) microarray technology, an increasing number of large rare deletions have been detected in schizophrenia patients^8–10^. However, the effect size associated with common CNVs is smaller than initially estimated^11^. In addition, many candidate genes for schizophrenia have been found by genome-wide association studies (GWAS)^12–14^. However, these SNPs are at frequencies of 20–80% in the general population and only account for a minimal increase in risk^15^. It has been shown that many complex traits or diseases including schizophrenia are driven by an accumulation of enormously large numbers of variants of small effects^14, 16–19^.

An allele can belong to either the major or the minor allele according to its frequency in the population and the minor allele (MA) has frequency (MAF) <0.5. Most known risk alleles are MAs^20^. Our previous studies have shown that the collective effects of genome wide MAs may play a role in numerous traits and diseases^21–23^. Specifically, enrichment of genome wide common SNPs or MAs is associated with Parkinson’s disease (PD)^21^ and lower reproductive fitness in *C*.*elegans* and yeasts^22^. To further explore these intriguing observations, we here studied the role of genome wide MAs as a collective whole in schizophrenia using previously published GWAS datasets and performed risk prediction using a selected set of MAs.

## Results

### Accumulation of minor alleles in schizophrenia

We made use of the published GWAS datasets (GAIN and MGS)^12, 19, 24, 25^. We first cleaned these datasets by removing outliers in Principal component analysis (PCA) plots (Supplementary Fig. S1). The cleaned datasets contained 1 002 cases and 1 152 controls in GAIN cohort, and 827 cases and 1 068 controls in MGS cohort. MA status of each SNP was then obtained by using the control cohort with MAF < 0.5 as cutoff. Minor allele content (MAC) of each subject was next calculated (total number of MAs per subject divided by the total number of SNPs analyzed), and the mean MAC values of cases and controls were compared. For the complete set of cleaned SNPs (total SNPs after quality control [QC], 696 460 SNPs), the mean MAC of schizophrenia cases was significantly higher than that of controls in both the GAIN data (*P* = 9.83E-09, z-test, Table 1) and the MGS data (*P* = 6.46E-04, z-test, Table 1). In addition, we pruned SNPs with linkage disequilibrium (LD) analysis using different pairwise r^2^ threshold (0.8, 0.7, 0.6, 0.5, 0.4, 0.3, 0.2 and 0.1), and obtained different sets of LD-independent SNPs. We combined subjects in the two cohorts for analyzing these LD-independent SNPs out of sample size considerations and recalculated MAC values accordingly. Again, MAC was found to be significantly different between cases and controls for all different sets of LD-independent SNPs examined (z-test, Table 1). These results indicated genome wide MAs enrichment in schizophrenia.

**Table 1:** MAC values in cases and controls calculated from either total SNPs (after QC) or LD-independent SNPs of different r^2^ threshold.

Subjects (controls:cases)	SNPs set	NO. SNPs	MAC (mean ± S.E.M.)		P-value
			controls	cases	
GAIN (1152:1002)	total SNPs	696 460	0.23577 ± 3.20E-05	0.23603 ± 3.29E-05	9.83E-09
MGS (1068:827)	total SNPs	696 460	0.23577 ± 3.25E-07	0.23594 ± 3.79E-05	6.46E-04
GAIN + MGS (2220:1829)	r^2^ > 0.8	337 589	0.22213 ± 1.95E-05	0.22224 ± 2.09E-05	2.06E-04
	r^2^ > 0.7	286 497	0.21584 ± 1.92E-05	0.21594 ± 2.07E-05	4.49E-04
	r^2^ > 0.6	240 608	0.20776 ± 1.90E-05	0.20785 ± 2.06E-05	2.21E-03
	r^2^ > 0.5	198 495	0.19771 ± 1.93E-05	0.19782 ± 2.07E-05	9.72E-05
	r^2^ > 0.4	157 339	0.18365 ± 1.95E-05	0.18374 ± 2.09E-05	3.67E-03
	r^2^ > 0.3	119 326	0.16462 ± 2.05E-05	0.16470 ± 2.19E-05	9.80E-03
	r^2^ > 0.2	82 774	0.13919 ± 2.21E-05	0.13930 ± 2.40E-05	6.76E-04
	r^2^ > 0.1	44 459	0.10535 ± 2.53E-05	0.10550 ± 2.80E-05	5.35E-05

S.E.M.: Standard Error of the Mean.

**Figure 1:**
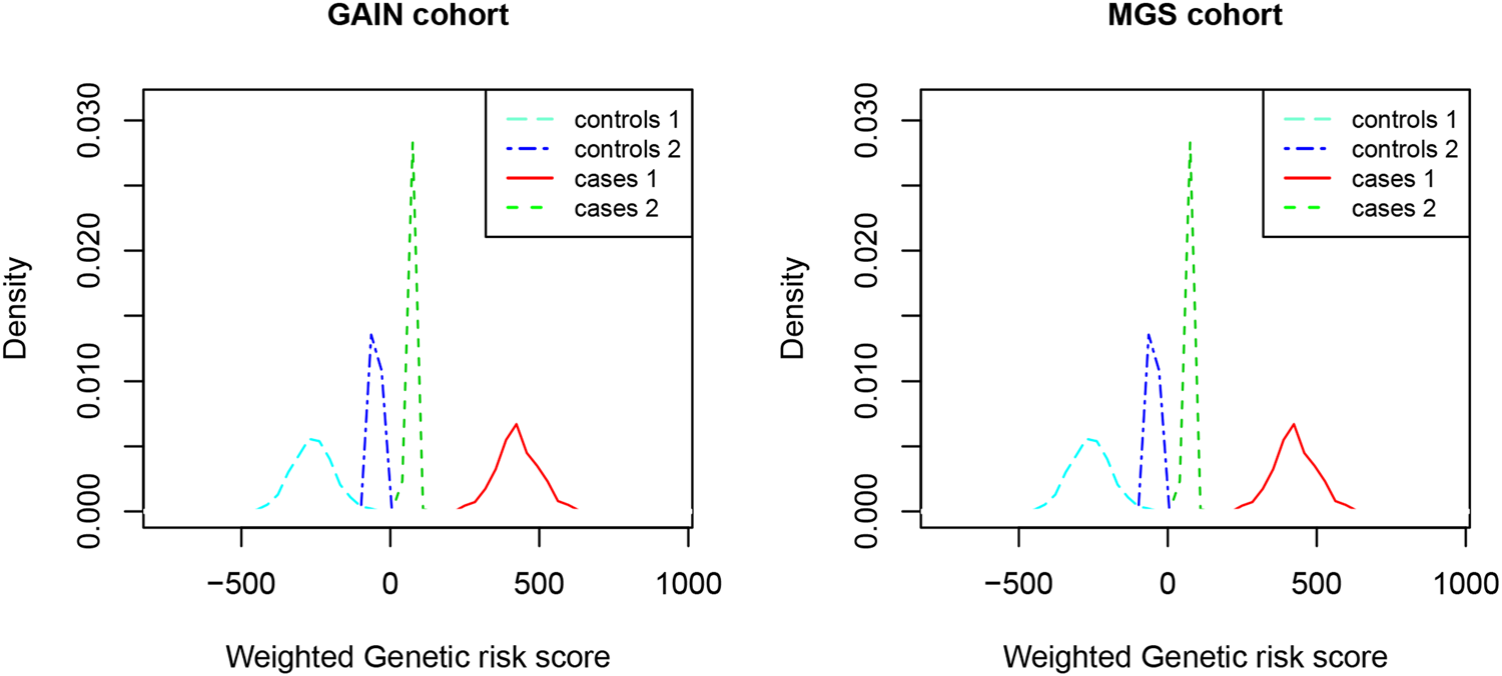
Weighted genetic risk score distribution in cases and controls. Distribution of weighted genetic risk score with total SNPs and LD-independent SNPs of case and control subjects in GAIN and MGS cohort. Controls 1 and cases 1: calculated with total SNPs; controls 2 and cases 2: calculated with LD-independent SNPs with r^2^ threshold of 0.3.

We then calculated a risk coefficient score for each SNP by logistic regression analysis and obtained a weighted genetic risk score (wGRS) based on the MA status and the risk coefficient score as previously described^21^. The MAC of each individual was then converted into a weighted risk score by summing up the weighted risk scores of each SNP. The mean wGRS of cases was found to be far greater than that of controls in both datasets when analyzed using the total SNPs (Fig. 1, mean wGRS [mean ± SEM] in GAIN cohort, cases [n = 1 002] 425.52 ± 2.23 vs controls [n = 1 152] −261.22 ± 2.07, *P* < 0.001; for MGS cohort, cases [n = 827] 392.31 ± 2.69 vs controls [n = 1068] −388.37 ± 2.45, *P* < 0.001, z-test). For LD-independent SNPs, we only compared wGRS between cases and controls using SNPs with r^2^ threshold of 0.3, because the MAC difference between cases and controls in this set of SNPs was the smallest (P value was the largest) among all LD-independent SNPs sets (so, if this set showed meaningful and positive results, other sets with smaller P-values would be expected to show the same). The results showed that the wGRS of LD-independent SNPs with r^2^ 0.3 was higher in cases than in controls (Fig. 1, cases [n = 1 002] 68.59 ± 0.32 vs controls [n = 1 152] −47.67 ± 0.30 in GAIN cohort, *P* < 0.001; cases [n = 827] 69.36 ± 0.39 vs controls [n = 1068] −63.48 ± 0.34 in MGS cohort, *P* < 0.001, z-test). This was apparent on a density plot of the wGRS with clearly separated cases and controls using both total SNPs and LD-independent SNPs with r^2^ threshold of 0.3 in GAIN and MGS cohort (Fig. 1).

### Evaluation of wGRS models in risk prediction

**Figure 2:**
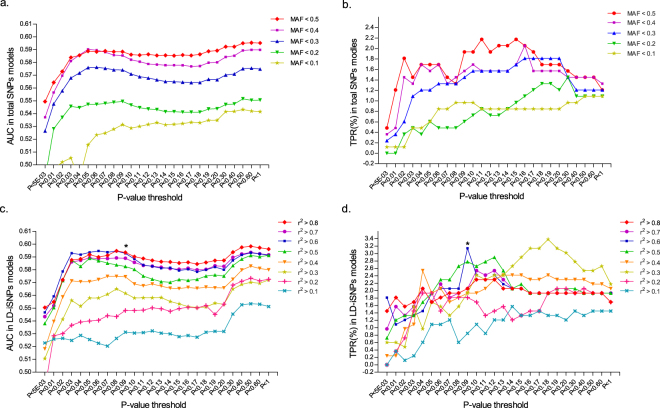
Discriminatory abilities of different wGRS prediction models from external cross-validation analysis. Discriminatory abilities of 130 wGRS prediction models constructed by total SNPs (**a**,**b**). Discriminatory abilities of 208 wGRS prediction models constructed by LD-independent SNPs (**c**,**d**). AUC (**a**,**c**) and TPR (**b**,**d**) were calculated using a training dataset (GAIN) and a validation dataset (MGS) to evaluate the discriminatory abilities. *The optimal model with the best performance among models constructed by LD-independent SNPs.

We next performed risk prediction using wGRS constructed from MAs of both total SNPs and LD-independent SNPs. In order to get an optimal amount of MAs for prediction of schizophrenia from an independent case-control blind database, we constructed 338 models using total SNPs or LD-independent SNPs for risk prediction. For total SNPs, we made 130 prediction models based on 5 different MAF cutoffs and 26 different P-values of logistic regression analysis (Fig. 2a,b and Supplementary Table S1). For LD-independent SNPs, we made 208 prediction models based on 8 different r^2^ thresholds of LD analysis (with all SNPs used for model construction having MAF < 0.5) and 26 P-values of logistic regression analysis (Fig. 2c,d and Supplementary Table S2). We then performed external cross-validation and internal cross-validation analyses to test these models. In external cross-validation, we used the GAIN cohort as the training dataset and the MGS cohort as the validation dataset. We used the receiver operator characteristic (ROC) curve (or area under the curve [AUC] of each model in the validation dataset) and true positive rate (TPR) to examine the discriminatory capability. The results showed good discriminatory capability using models constructed with both LD-independent SNPs and total SNPs (Fig. 2 and Supplementary Tables S1 and S2).

To further evaluate the accuracy of those models as shown in Fig. 2 that performed well in external cross validations (TPR >= 2% and AUC > 0.57 in total SNPS models, or TPR >= 2.78% and AUC > 0.57 in LD-independent SNPs models), a 10 fold internal cross-validation analysis^26^ was performed using the GAIN cohort. Each model was analyzed 10 times, and the mean AUC and TPR values were calculated. Based on both external and internal cross-validation analyses, the best model using total SNPs was found to have AUC 0.5857 (95% CI, 0.5599–0.6115) and TPR 2.18% (95% CI, 1.295–3.418%) in external cross-validation analysis, and AUC 0.6017 (95% CI, 0.5779–0.6254) and TPR 3.78% (95% CI, 1.650–5.907%) in internal cross-validation analysis. There were 82 925 SNPs in this model with MAF < 0.5 and each MA with a *P* < 0.11 (external cross-validation analysis results see Fig. 2a,b and Supplementary Table S1, internal cross-validation results see Supplementary Table S1). For the LD-independent SNPs, the best model was found by using SNPs with r^2^ threshold of 0.6 and *P* < 0.09 (MAF < 0.5), which had AUC 0.5928 (95% CI, 0.5672–0.6185) and TPR 3.14% (95% CI, 2.064–4.573%) in external cross-validation analysis, and AUC 0.6153 (95% CI, 0.5872–0.6434) and TPR 3.26% (95% CI, 1.263–5.263%) in internal cross-validation analysis. This model contains 23 238 SNPs (external cross-validation analysis results see Fig. 2c,d and Supplementary Table S2, internal cross-validation results see Supplementary Table S2).

We also evaluated the capacity of wGRS to predict case-control status using the Nagelkerke’s method, a likelihood-based measure to quantify the goodness-of-fit of models containing genetic predictors of human disease^14, 19, 27^. For this analysis, we analyzed the models with good performance in the cross validation analysis (Table 2). The variance explained of Nagelkerke’s R^2^ value (from external cross-validation analysis) was 3.99% for the best model from total SNPs and 4.61% for the best model from LD-independent SNPs. Based on the above evaluation results, we chose the best model from LD-independent SNPs as the optimal model for subsequent analysis, which had higher TPR, AUC and Nagelkerke’s R^2^ value and with less number of SNPs.

**Table 2:** The variance explained of Nagelkerke’s - R^2^ (%) in MGS cohort based on weighted Genetic Risk Scores (wGRS).

SNPs set	P threshold	R^2^
Total SNPs	0.15	3.97
	0.13	3.97
	0.11	3.99
r^2^ > 0.8	0.12	4.02
	0.11	4.05
	0.10	4.09
r^2^ > 0.7	0.12	3.80
	0.11	3.82
	0.10	3.91
r^2^ > 0.6	0.12	3.82
	0.10	4.24
	0.09	4.61
r^2^ > 0.5	0.12	3.13
	0.09	3.68
	0.08	3.76
r^2^ > 0.4	0.17	2.50
	0.15	2.46
	0.14	2.43
r^2^ > 0.3	0.20	1.88
	0.18	1.85
	0.16	1.83

wGRS analyses using MGS samples as validation cohort and GAIN samples as training cohort. Either total SNPs or LD-independent SNP sets of different r^2^ values (threshold of LD analysis) as indicated were used for the analysis of R^2^ values representing variance explained by Nagelkerke’s method. Only the models with good performance of AUC and TPR value in cross-validation analyses were analyzed.

### Comparison wGRS models to polygenic risk scores models

Previous studies showed that polygenic risk scores (PRS) constructed from common variants of small effects can predict case-control status in schizophrenia^19^. To compare the PRS method with our wGRS approach, we performed external-cross validation analysis by constructing PRS models using the GAIN and MGS cohorts. The same as the wGRS models, 9 SNPs sets were used including 1 total SNPs sets (after QC) and 8 LD-independent SNPs sets, and 26 models for each SNPs set were constructed based on P-values of logistic regression analysis, thus resulting in a total of 234 PRS models (all SNPs with MAF < 0.5). The GAIN cohort was used as the training data and the MGS as the validation data in the external cross-validation analysis. PRS calculation of each subject, PRS models construction and cross-validation analyses were performed with PRSice software^28^. AUC, TPR and variance explained of Nagelkerke’s R^2^ value of each model were calculated to measure the discriminatory abilities (Supplementary Fig. S2 and Supplementary Table S3). The model with the largest TPR value contained 31 107 SNPs with r^2^ threshold of 0.7 and *P* < 0.12, and had AUC 0.5792 (95% CI, 0.5534–0.6051), TPR 3.02% (95% CI, 1.966–4.430%) and variance explained of Nagelkerke’s R^2^ value 3.46%. The model with the largest AUC and Nagelkerke’s R^2^ value was from the total SNPs set with *P* < 0.6 (containing 359 089 SNPs) and had AUC 0.5935 (95% CI, 0.5678–0.6192), TPR 1.45% (95% CI, 0.7519–2.521%) and Nagelkerke’s R^2^ 4.33% (Supplementary Fig. S2 and Supplementary Table S3). The prediction capacities of these two PRS models were both slightly worse than the optimal wGRS model, which had AUC 0.5928, TPR 3.14%, and Nagelkerke’s R^2^ 4.61%.

### Prediction performance of different types of SNPs

We next examined the potential functions of the 23 238 SNPs in the optimal wGRS model by annotating them with the ANNOVAR software^29^, and compared the prediction results of different types of SNPs to that of total SNPs (after QC). Relative to total SNPs, the proportion of SNPs in the optimal wGRS model in exonic, upstream-downstream, and UTR regions were increased ([total vs optimal] exonic, 0.88% vs 1.14%, *P* = 4.20E-05; upstream-downstream, 1.09% vs 1.36%, *P* = 1.33E-04; UTR 1.02% vs 1.17%, *P* = 0.033, chi-square test, Supplementary Table S4), indicating an enrichment in gene coding and gene regulatory regions. No significant changes were found in other regions.

**Figure 3:**
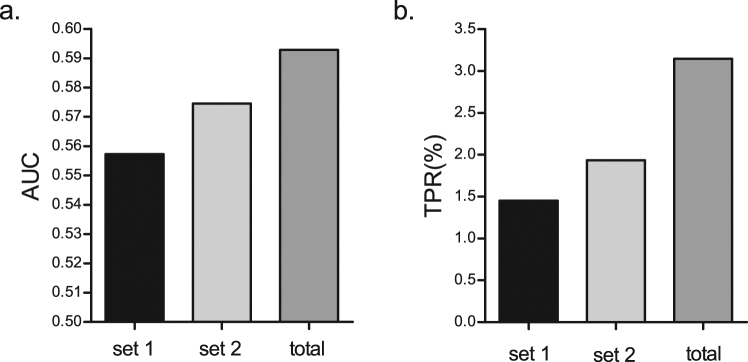
Role of intergenic SNPs in prediction performance. (**a**) AUC values. (**b**) TPR values. set1: without intergenic SNPs in optimal wGRS models; set 2: intergenic SNPs in optimal wGRS models; total: all SNPs contains in optimal wGRS models.

More than half of all SNPs in the optimal wGRS model were found located in intergenic regions (53.34%). We next divided the SNPs in the optimal model into two groups, one containing only intergenic SNPs and the other containing all SNPs except intergenic SNPs, and did external-cross validation analysis of risk prediction with both groups and compared them to the optimal model. We found that the intergenic SNPs group produced better AUC and TPR than that without the intergenic SNPs and both groups were worse than the optimal model in AUC and TPR values (Fig. 3). Thus, MAs in intergenic regions are an important component in the overall collective effect of MAs.

### Ontology and pathway analyses

**Figure 4:**
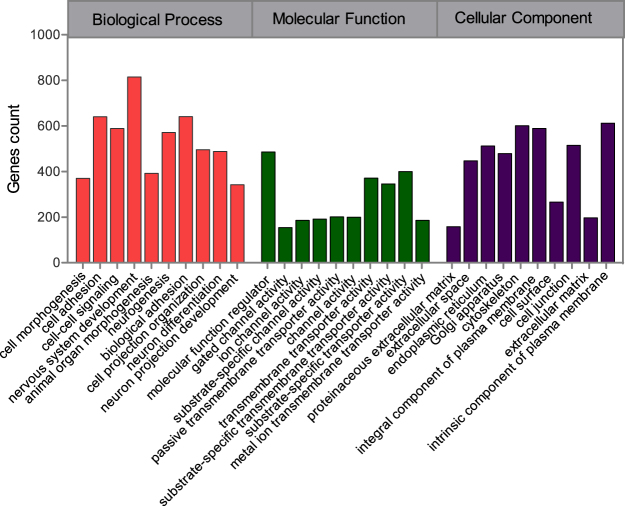
Top-10 enriched gene modules of gene ontology analysis with SNPs in optimal weighted Genetic Risk Scores model. Significant enrichment of gene modules was analyzed from WebGestaltR based on categories of biological process, molecular function, and cellular component respectively, more details can be found in Supplementary Table S6-1.

We mapped the 23 238 SNPs in the optimal wGRS model to gene loci using WebGestaltR tools, and found 16 135 SNPs unambiguously mapped to 6 255 unique entrez gene IDs (entire data see Supplementary Table S5). These genes were characterized using gene ontology in WebGestaltR according to biological process, molecular function, and cellular component. As shown in Fig. 4 (more details see Supplementary Table S6-1), the top-10 enriched genes modules were related to molecular function regulator, channel activity, and transporter activity. In terms of biological process, certain nervous system development, neurogenesis, neuron differentiation and neuron projection development genes were present, which have been linked with schizophrenia.

Pathway analysis was carried out on these 6 225 unique entrez gene IDs according to the KEGG using WebGestaltR tools. A total of 30 pathways were identified with false discovery rate (FDR) < 0.02 and had significant P values (after Benjamini-Hochberg adjustment) (Table 3 and Supplementary Table S6-2). Among these, 23 were found to be involved in one or more of the following known to be related to schizophrenia, such as axon guidance^30^, Rap1 signaling pathway^31, 32^, Glutamatergic synapse^33^, ECM-receptor interaction^34, 35^, focal adhesion^36^, PI3K-Akt signaling pathway^37^, retrograde endocannabinoid signaling^38^, Ras signaling pathway^32^, cell adhesion molecules (CAMs)^39^, morphine addiction^40^, GABAergic synapse^41^, neuroactive ligand-receptor interaction^42^, MAPK signaling pathway^43^, Hedgehog signaling pathway^44^, oxytocin signaling pathway^45^, circadian entrainment^46^, nicotine addiction^47^, long-term depression^48^, calcium signaling pathway^49^, inflammatory mediator regulation of TRP channels^50^, adherin junction^51^, regulation of actin cytoskeleton^52^, and cholinergic synapse^53^. The remaining 7 pathways may also play some roles in brain disorders including schizophrenia. Arrhythmogenic right ventricular cardiomyopathy (ARVC) pathway is related to cardiovascular disease, and has been found abnormal in schizophrenia patients^54^. Taste receptor expression in the dorsolateral prefrontal cortex in chronic schizophrenia is reduced, implicating taste transduction pathway in schizophrenia^55^. Pathways in cancer and Melanoma may be related to both cancer and neurological diseases as there are reports of a reduced risk of cancer among individuals with PD^56^ and Alzheimer’s disease (AD)^57^ and an increased risk of malignant melanoma associated with a PD diagnosis^58^. AGE-RAGE signaling pathway in diabetic complications has been implicated in the pathogenesis of diverse diseases including neurological disorders^59, 60^. The vascular smooth muscle contraction pathway is related to epilepsy^61^. Phospholipase D is related to metabolic diseases and may be a therapeutic target in certain brain disorders^62^.

**Table 3:** Significantly enriched KEGG pathways from WebGestaltR with SNPs in optimal weighted Genetic Risk Scores model.

ID	Pathways	#Gene	FDR	P-value
hsa04360	Axon guidance	90	2.32E-06	7.65E-09
hsa04015	Rap1 signaling pathway	99	4.58E-06	3.02E-08
hsa04724	Glutamatergic synapse	59	1.56E-05	1.54E-07
hsa04512	ECM-receptor interaction	47	2.41E-05	3.18E-07
hsa04510	Focal adhesion	96	3.74E-05	6.16E-07
hsa05200	Pathways in cancer	163	1.82E-04	3.60E-06
hsa04151	PI3K-Akt signaling pathway	137	1.99E-04	4.65E-06
hsa04723	Retrograde endocannabinoid signaling	52	1.99E-04	5.27E-06
hsa04014	Ras signaling pathway	96	3.41E-04	1.01E-05
hsa05412	Arrhythmogenic right ventricular cardiomyopathy (ARVC)	41	3.90E-04	1.29E-05
hsa04514	Cell adhesion molecules (CAMs)	67	6.84E-04	2.48E-05
hsa05032	Morphine addiction	46	9.78E-04	3.87E-05
hsa04727	GABAergic synapse	44	1.17E-03	5.04E-05
hsa04080	Neuroactive ligand-receptor interaction	110	1.69E-03	7.81E-05
hsa04010	MAPK signaling pathway	103	3.79E-03	1.88E-04
hsa04933	AGE-RAGE signaling pathway in diabetic complications	49	5.64E-03	2.98E-04
hsa04340	Hedgehog signaling pathway	26	8.34E-03	4.90E-04
hsa04921	Oxytocin signaling pathway	67	8.34E-03	4.95E-04
hsa05218	Melanoma	35	8.36E-03	5.34E-04
hsa04270	Vascular smooth muscle contraction	53	8.36E-03	5.52E-04
hsa04713	Circadian entrainment	44	8.60E-03	5.96E-04
hsa05033	Nicotine addiction	23	9.23E-03	6.93E-04
hsa04742	Taste transduction	33	9.23E-03	7.01E-04
hsa04072	Phospholipase D signaling pathway	61	1.03E-02	8.67E-04
hsa04730	Long-term depression	31	1.03E-02	9.20E-04
hsa04020	Calcium signaling pathway	74	1.03E-02	9.45E-04
hsa04750	Inflammatory mediator regulation of TRP channels	45	1.03E-02	9.46E-04
hsa04520	Adherens junction	36	1.03E-02	9.48E-04
hsa04810	Regulation of actin cytoskeleton	87	1.38E-02	1.32E-03
hsa04725	Cholinergic synapse	47	1.66E-02	1.65E-03

P-values were adjusted with the Benjamini-Hochberg method. Only pathways with FDR < 0.2 and of significant p-value were included, more details can be found in Supplementary Table S6-2.

## Discussion

In this study, we showed enrichment of MAs in schizophrenia cases relative to matched controls, suggesting a role for the collective effects of polygenic variation in the risk for schizophrenia. We also calculated wGRS of each subject based on MA status of SNPs and did risk prediction using wGRS. We identified a set of MA of common SNPs that can specifically predict a fraction of schizophrenia cases. We further showed that SNPs located in the gene coding and gene regulatory regions were enriched in the optimal prediction model but SNPs located in the intergenic region were also important for the overall collective effect of MAs.

Polygenic inheritance of complex traits and diseases has long been hypothesized^63^. The PRS method was first applied by the International Schizophrenia Consortium to evaluate the aggregation effect of polygenic variation in Schizophrenia^19^. The method has since been used in other complex traits and diseases. Different from the PRS method’s focus on disease-associated SNPs, the wGRS method we developed here considered all minor alleles on a genome wide scale. The main difference between the wGRS and PRS model construction was the calculation of total risk scores of each individual. The PRS of each individual was obtained by summing up weighted log_10_(odds ratio) of disease-associated alleles (odds ratio obtained from logistic regression tests). These alleles were weighted by effect sizes estimated from a genome-wide association study^19, 28^. The wGRS of each individual was obtained by summing up weighted beta regression coefficient of each SNP based on MA status of SNPs with beta regression coefficient calculated by logistic regression tests. Different p-value thresholds were used in both PRS and wGRS models construction. Our direct comparison of these two methods found the wGRS method to be slightly better.

We found that LD pruning can increase the prediction accuracy in wGRS prediction analysis. Similar to our previous method (models based on different MAF and P-value with total SNPs) to predict PD^21^, we obtained a large set of 82 925 MAs of SNPs from a total set of 696 460 SNPs that can predict 2.18% schizophrenia (AUC 0.5857) or explaining 3.99% of the phenotype variance (Table 2). However, the best model from LD pruned SNPs had TPR value increased to 3.14% (AUC 0.5928), the explained variance increased to 4.61%, and SNPs numbers decreased to 23 238.

Several measures may be considered to increase prediction accuracy in the future. Increasing sample size has been shown to an effectively way. The Nagelkerke’s -R^2^ value increased from 3.4%^19^ to 18.4%^14^ along with data size increased from 2 176 cases/1 642 controls to 32 838 cases/44 375 controls. In addition, integrating informations such as clinical features^64^, pleiotropy^65^ and functional annotations^66^, joint modeling of correlated traits^67^ could also improve prediction accuracy for complex diseases.

There were reports of male bias in schizophrenia^68^. The ratio of males to females in cases of GAIN cohort was 2.27 (696:306) and 2.18 (567:260) in the MGS cohort. We however did not observe significant differences in MAC values between male and female cases in both datasets (Supplementary S7).

Recent studies have shown that a much larger than expected portion of the human genome may be functional^69, 70^. Our study here is consistent with this as more than half of SNPs in the optimal wGRS model we identified here were located in intergenic regions, which were critical to our prediction model. The enrichment of risk SNPs in the gene coding and gene regulatory regions as found here is to be expected given that these regions are known to have greater functional effects, which also served to validate our approach here.

Most of the enriched pathways found here were known to relate to schizophrenia and other neurological disorders. It should be noted that these pathways and the ontology results were obtained by using SNPs from the optimal wGRS model. It is possible that different SNP sets from different models may identify different pathways and different genes modules. In addition, all subjects we used in this study were of European ancestry and it remains to be seen whether similar findings could be replicated in other racial groups.

Genetic diversities today are clearly at saturation levels as indicated by the observation that higher fractions of fast evolving SNPs, relative to slow evolving ones, are shared between different human groups^71, 72^. This raises the question of what selection forces are keeping genetic diversity levels from increasing with time. By linking the total amount of SNPs or MAs in an individual to complex diseases and traits, it is clear that complex diseases could serve as a negative selection mechanism to prevent abnormal increase in SNP numbers in an individual^73^. It is expected that the overall property of the genome as a whole should be linked with the wellbeing of an organism. Our results here on schizophrenia further confirmed the hypothesis we put forward before that a highly complex and ordered system such as the human brain must have an optimum limit on the level of randomness or entropy in its building parts or DNAs^21^.

Using LD-independent SNPs, we identified a set of 23 238 MAs that could predict 3.14% cases specifically. The value is similar to 22q11.2 deletion, which accounts for approximately 1~2% of all cases of schizophrenia^5, 74^. These SNPs were linked with pathways known to be involved in the disease, thereby validating our method of looking for disease specific set of SNPs. This set is larger than any known from previous studies^19^. Future studies using larger sample sizes and integrating additional information may help identify a more specific set of risk SNPs that could improve prediction performances.

## Materials and Methods

### Subjects

We included two GWAS datasets of cases and controls in our analysis, GAIN (phs000021.v3.p2) and MGS (phs000167.v1.p1)^12, 19, 24, 25^. Both datasets were downloaded from database of Genotypes and Phenotypes (dbGaP). All subjects we selected for analysis are European ancestry population. There were no any overlap individuals between two datasets. Whole genome genotyping of subjects was scanned with AFFY_6.0 of Affymetrix. PCA using the GCTA tool was performed to analyze the genetic homogeneity of the subjects^75^. There were three principal component (PC) factors generated based on the genotypes of each subject from analysis, subjects with similar PC values were kept, outliers were excluded if PC values of individuals has large difference compared with other individuals (more details see Supplementary Fig. S1).

### SNPs selection

All SNPs for analysis in this study are autosomal SNPs. In addition, genotype data of each individual were subjected to rigorous QC measures to exclude poor-quality SNPs^21^. Therefore, we excluded SNPs showing departure from the Hardy-Weinberg equilibrium (P < 0.01), with missing data <5%, and with MAF < 0.01. The removal of rare alleles was meant to eliminate any artefactual effects by rare SNPs that might be misidentified due to errors. After these filters, there were 696 460 SNPs remaining (Table 1).

For the different sets of LD-independent SNPs, we used Plink to prune SNPs according to different pairwise r^2^ threshold (0.8, 0.7, 0.6, 0.5, 0.4, 0.3, 0.2 and 0.1 respectively) within a 200 kb window. The numbers of remaining SNPs after pruning were presented in Table 1.

### Statistical analysis

The Hardy-Weinberg equilibrium, missing data, MAF, LD and logistic regression analysis were performed using PLINK Tools^76^. MAC of each subject was obtained using total number of MAs divided by the total number of SNPs scanned (non-informative SNPs were excluded). The script for MAC calculation was previously described^21^. Risk coefficient (beta regression coefficient) of each SNP was calculated with logistic regression test (equal to coefficient logistic regression test). The wGRS of a MA was calculated as follows: for homozygous MA, the risk coefficient was 1 x the coefficient, for heterozygous MA, it was 0.5 x the coefficient, for homozygous major allele, the coefficient was 0. The total wGRS from all MAs in a subject was obtained by summing up the weighted risk coefficient of all MAs by the script as described previously^21^. Before comparison of mean MAC and wGRS differences of cases and controls, F-test in excel was used to test homogeneity of variance of two groups. After confirming that all results show homogeneity of variance, z-test (two-tailed) in excel was performed to compare the mean MAC and wGRS between cases and controls. Chi-square test was used for comparison of two sample proportions with R software. The PRS calculation of each subject was done according to a previous study^19^ by summing up weighted log_10_(odds ratio) of each disease-associated SNP in a subject with odds ratio obtained from logistic regression tests. PRS calculation was performed using the PRSice software^28^.

### Construction and evaluation of genetic risk models

Models construction included wGRS models from total SNPs (after QC), wGRS models from LD-independent SNPs and PRS models from total and LD-independent SNPs. For wGRS models from total SNPs, all SNPs were divided into 5 groups according to MAF (MAF < 0.5, 0.4, 0.3, 0.2 and 0.1). Each group was further divided into 26 subgroups based on different p-value thresholds of logistic regression analysis (*P* < 1, 0.6, 0.5, 0.4, 0.3, 0.2, 0.19, 0.18, 0.17, 0.16, 0.15, 0.14, 0.13, 0.12, 0.11, 0.1, 0.09, 0.08, 0.07, 0.06, 0.05, 0.04, 0.03, 0.02, 0.01 and 0.005), resulting in a total of 130 models. For wGRS models from LD-independent SNPs, the SNPs were divided into 8 groups based on the r^2^ threshold (r^2^ > 0.8, 0.7, 0.6, 0.5, 0.4, 0.3, 0.2, 0.1), with each group further divided into 26 subgroups based on different p-value thresholds as above, resulting in a total of 208 models. All SNPs in these models had MAF < 0.5. For PRS models construction, all SNPs were divided to 9 groups (1 total SNPs group and 8 different r^2^ threshold groups) with each group further divided into 26 subgroups based on different p-value thresholds, resulting in a total of 234 models (all SNPs with MAF < 0.5).

To evaluate the wGRS models, external cross-validation and internal cross-validation were performed and AUC, TPR and Nagelkerke’s - R^2^ values of models were calculated to evaluate the ability to differentiate cases and controls. For external cross-validation, the GAIN cohort was used as training dataset, and the MGS cohort as validation dataset. For the internal cross-validation, a 10 fold cross-validation^26^ was used to test the models with good performance in external cross-validation. Subjects in GAIN cohort were divided into 10 sub-sets randomly. For randomly assigning a subject to a group, all subjects were assigned a value randomly generated using the function RAND () in excel, and then sorted according to the value. This list was then equally divided into 10 sub-sets with ~216 subjects each (4 sub-sets with 216 subjects and 6 with 215 subjects). When a sub-set was used as the validation data, the other 9 sub-sets together were used as the training data. The cross-validation process was repeated 10 times, and the mean AUC and TPR values were calculated from these 10 results. The model with the largest AUC, TPR as well as Nagelkerke’s -R^2^ value was selected as the best (optimal) model for subsequent analysis. If two models have similar values, the model with a smaller number of SNPs was selected as the best.

To evaluate the PRS models, external cross-validation was performed using the PRSice software^28^. The GAIN cohort was used as the training dataset and MGS cohort as the validation dataset. AUC, TPR and Nagelkerke’s - R^2^ values of each model were calculated to evaluate the ability to differentiate cases and controls.

AUC values for each model were calculated by R with ‘pROC’ packages^77^. TPR is the proportion of cases with wGRS or PRS higher than all of the controls, with 100% specificity, and was calculated by GraphPad Prism5. Nagelkerke’s - R^2^ values (obtained from logistic regression analysis) were used to estimate the proportion of variance explained by wGRS or PRS. The number of SNPs used to calculate the wGRS or PRS per individual was recorded as a covariate. Variance explained of Nagelkerke’s - R^2^ was calculated as the Nagelkerke’s - R^2^ value of the model including wGRS and covariates minus that of the model including only covariates.

### SNPs annotation and functional enrichment analyses

ANNOVAR (http://annovar.openbioinformatics.org/) was used to annotate SNPs^29^. For functional enrichment analysis, WebGestaltR (http://bioinfo.vanderbilt.edu/webgestalt/) tools were used for gene ontology annotation and pathway analysis based on Kyoto Encyclopedia of Genes and Genes (KEGG) (http://www.genome.jp/kegg/)^78, 79^.

## Electronic supplementary material

Supplementary Table S1

Supplementary Table S2

Supplementary Table S3

Supplementary Table S5

Supplementary Table S6

Supplementary information
