# Supplementary material for: Accumulation of minor alleles and risk prediction in schizophrenia
Source: Sci Rep. 2017 Sep 15;7:11661. doi: 10.1038/s41598-017-12104-0 (PMC5601945; doi:10.1038/s41598-017-12104-0)
Supplement: Supplementary file 6 — Supplementary information [file 41598_2017_12104_MOESM6_ESM.pdf]

SUPPLEMENTARY INFORMATION

Accumulation of minor alleles and risk prediction in Schizophrenia

Pei He[1], Xiaoyun Lei[1], Dejian Yuan[1], Zuobin Zhu[2], and Shi Huang[1]*

[1] Laboratory of Medical Genetics, School of life sciences, Central South University, 110 Xiangya Road, Changsha, Hunan, 410078, China

[2] Department of Genetics, Xuzhou Medical University, Xuzhou, Jiangsu 221004, China.

* Corresponding author : huangshi@sklmg.edu.cn

Supplementary Fig S1. Principal component analysis (PCA) of subjects of European Americans. The subjects were analyzed using the GCTA tool. Three principal component (PC) factors were generated from analysis, pc1, pc2, pc3. Scatter plots were drawn respectively based on pc1 and pc2 (a) or pc2 and pc3 (c). We excluded the subjects outside the boxed area in panels a and c. The subjects that remained after the filtering were analyzed by PCA plot again (b and d), and contained. 1 002 cases and 1 152 controls in GAIN cohort (phs21), and 827 cases and 1 068 controls in MG cohort (phs167).

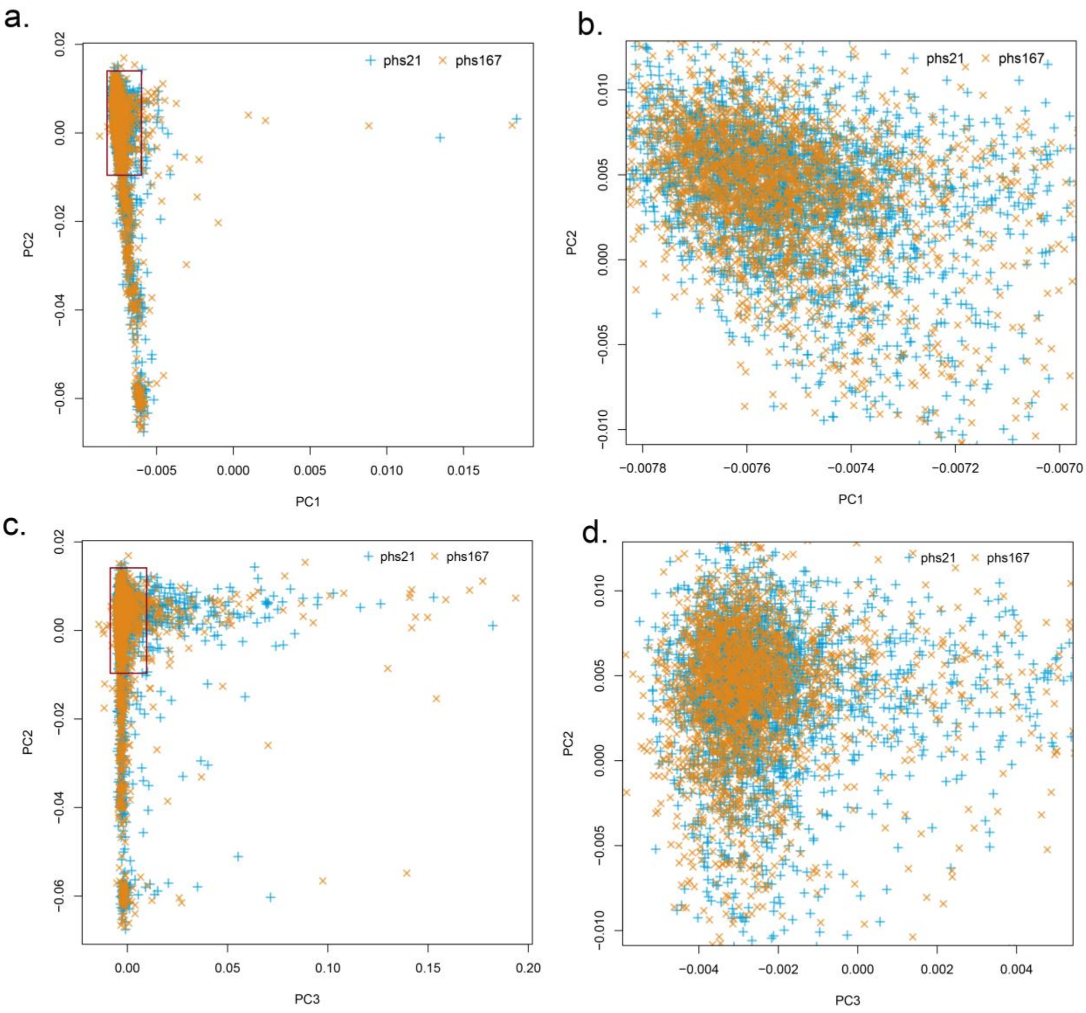

Supplementary Table S1. Summary of AUC and TPR values of wGRS models using total SNPs in external cross-validation and internal cross-validation.

Supplementary Table S2. Summary of AUC and TPR values of wGRS models using LD-independent SNPs in external cross-validation and internal cross-validation. All models had MAF < 0.5.

Supplementary Fig S2. Discriminatory abilities of different PRS prediction models from external cross validation analysis. Discriminatory abilities of 234 prediction models constructed by 1 total SNPs set and 8 LD-independent SNPs sets were analyzed by AUC (a), TPR (b), and the Nagelkerke's R^2 values (c). * : the model with the largest AUC and Nagelkerke's R^2 value. #： the model with the largest TPR value.

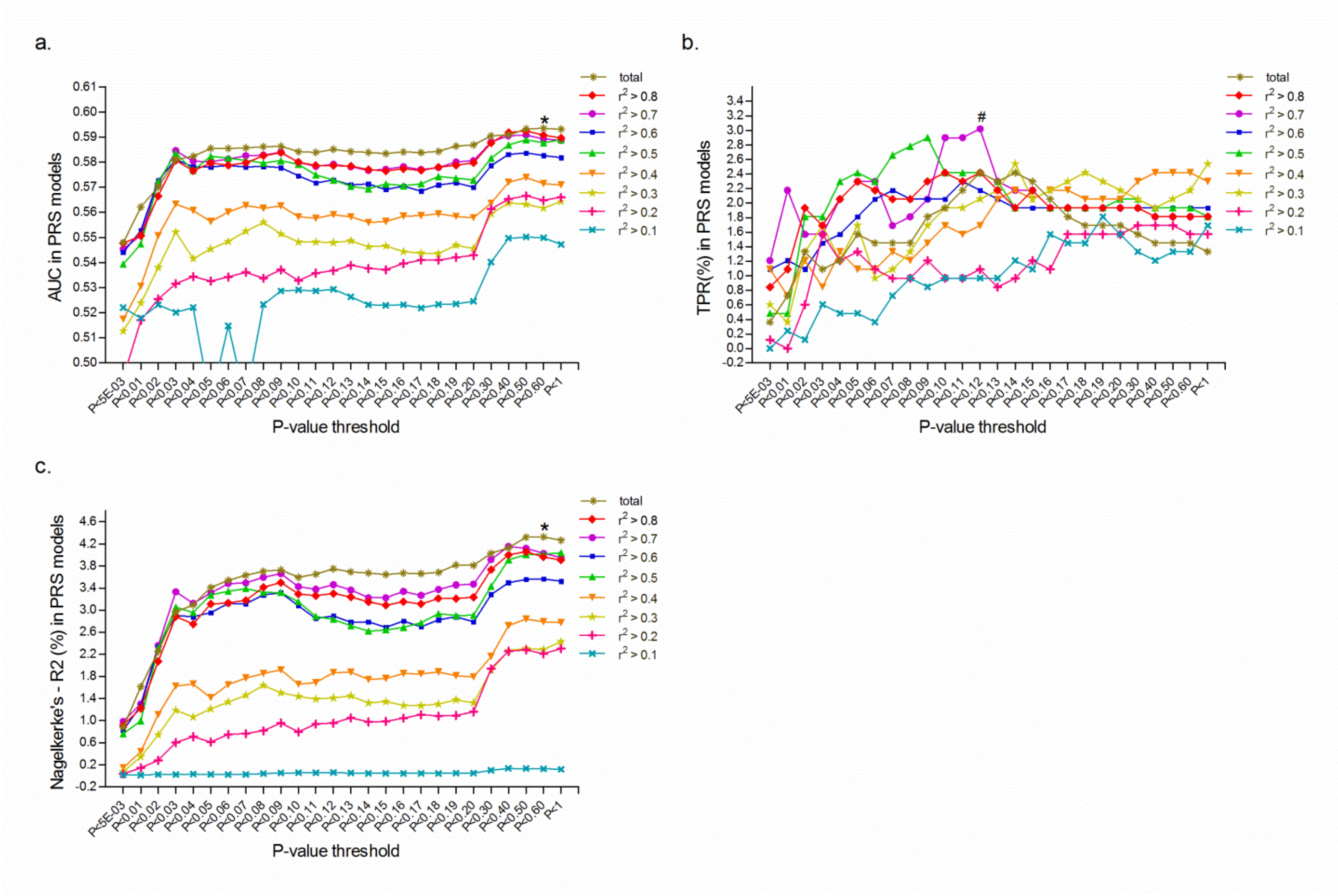

Supplementary Table S3. Summary of AUC and TPR and Nagelkerke's - R^2 values (variance explained) of PRS models in external cross-validation analysis. All models had MAF < 0.5.

Supplementary Table S4. Distribution profiles in different regions of the genome for SNPs. Shown are both the profile for the total SNPs set (696 460 SNPs after QC) and the profile for SNPs in the optimal wGRS prediction model (23 238 SNPs). SNPs were annotated with ANNOVAR software. The chi-square test was performed. *p-value was obtained from Fisher exact test. The numbers of SNPs from annotation results were somewhat different from those of the original numbers of SNPs, because there were some SNPs with multiple records due to multiple mapping, and certain variations ignored when annotating due to lack of correct annotation.

	total SNPs (atfer QC)		optimal model SNPs		p-value
	Number	percent%	Number	percent%	
intergenic	375075	53.83	12342	53.32	0.1234
intronic	259328	37.22	8560	36.98	0.4597
ncRNA_intronic	39020	5.60	1311	5.66	0.6917
upstream,downstream	7610	1.09	315	1.36	1.33E-04
UTR	7109	1.02	270	1.17	0.03255
exonic	6114	0.88	263	1.14	4.20E-05
ncRNA_exonic	2474	0.36	88	0.38	0.5658
splicing	36	0.01	0	0.00	0.6312*
Total	696766	100	23149	100	

Supplementary Table S5. SNPs in optimal wGRS model annotation results from WebGestalt information.

Supplementary Table S5-1. List of 16 135 SNPs from the best wGRS model mapped to 6 225 unique Entrez Gene IDs by WEB-based GEne SeT.

Supplementary Table S5-2. List of 7 103 SNPs in the best wGRS model not mapped by WEB-based GEne SeT AnaLysis Toolkit.

Supplementary Table S6. Enriched biological process, molecular function, cellular component, and KEGG pathways with SNPs in best wGRS model.

Supplementary Table S6-1. Top-10 enriched genes in categories of biological process, molecular function, and cellular component from WebGestaltR. C: the number of reference genes in the category; O: the number of genes in the gene set and also in the category; E: the expected number in the category; R: ratio of enrichment; P Value: adjusted by the Benjamini-Hochberg method.

Supplementary Table S6-2. Significantly enriched KEGG pathways from WebGestaltR. C: the number of reference genes in the category; O: the number of genes in the gene set and also in the category; E: the expected number in the category; R: ratio of enrichment; PValue: adjusted by the Benjamini-Hochberg method.

Supplementary Table S7. Summary and compare the average MAC value of male and female individuals in two dataset.

Supplementary Table S7-1. Summary the average MAC value of male and female

case or control individuals in two dataset.

	Total controls	Male controls	Female controls	Total cases	Male cases	Female cases
**GAIN**						
n subjects	1152	526	626	1002	696	306
mean MAC	0.235771	0.235761	0.235780	0.236035	0.236046	0.236009
S.E.M.	3.20E-05	4.60E-05	4.44E-05	3.29E-05	4.02E-05	5.74E-05
**MGS**						
n subjects	1068	534	534	827	567	260
mean MAC	0.235773	0.235772	0.235774	0.235943	0.235931	0.235970
S.E.M.	3.25E-05	4.87E-05	4.30E-05	3.79E-05	4.54E-05	6.87E-05

Supplementary Table S7-2. Comparation the difference of average MAC value among the groups of total controls, male controls, female controls, total cases, male cases and female cases in two datasets. The one-way ANOVA, Bonferroni's multiple comparison test was performed to compare the difference of MAC. There were no differences in MAC values between male and female cases or male and female controls in both datasets

		P-value	
**Group1**	**Group2**	**GAIN**	**MGS**
Total controls	Male controls	1.000	1.000
	Female controls	1.000	1.000
	Total cases	< 0.001	0.009
	Male cases	< 0.001	0.071
	Female cases	0.008	0.119
Male controls	Female controls	1.000	1.000
	Total cases	< 0.001	0.062
	Male cases	< 0.001	0.215
	Female cases	0.018	0.222
Female controls	Total cases	< 0.001	0.068
	Male cases	< 0.001	0.232
	Female cases	0.031	0.235
Total cases	Male cases	1.000	1.000
	Female cases	1.000	1.000
Male cases	Female cases	1.000	1.000
